# Supplementary material for: A Novel Ferroptosis-Related Gene Signature Predicts Overall Survival of Breast Cancer Patients
Source: Biology (Basel). 2021 Feb 14;10(2):151. doi: 10.3390/biology10020151 (PMC7917807; doi:10.3390/biology10020151)
Supplement: Supplementary file 1 [file biology-10-00151-s001.zip › final manuscript/table 3.docx]

**Table 3. Results of the** **univariate and multivariate Cox regression analyses regarding OS in the TCGA-BRCA derivation cohort and the METABRIC validation cohort**

| **Predictors** | **TCGA-BRCA** | | | | | | |  | **METABRIC** | | | | | | |
| --- | --- | --- | --- | --- | --- | --- | --- | --- | --- | --- | --- | --- | --- | --- | --- |
|  | Univariate analysis | | |  | Multivariate analysis | | |  | Univariate analysis | | |  | Multivariate analysis | | |
|  | HR | 95%CI | *P* |  | HR | 95%CI | *P* |  | HR | 95%CI | *P* |  | HR | 95%CI | *P* |
| **Age** | 1.032 | 1.02-1.045 | <0.001 |  | 1.035 | 1.022-1.048 | <0.001 |  | 1.036 | 1.03-1.041 | <0.001 |  | 1.054 | 1.046-1.062 | <0.001 |
| **Gender**  **(Male VS Female)** | 0.84 | 0.1168-5.993 | 0.859 |  | - | - | - |  | - | - | - |  | - | - | - |
| **Race** |  |  |  |  |  |  |  |  |  |  |  |  |  |  |  |
| Black (Ref) |  |  |  |  |  |  |  |  |  |  |  |  |  |  |  |
| White | 0.826 | 0.552-1.236 | 0.353 |  | - | - | - |  | - | - | - |  | - | - | - |
| Asian | 0.617 | 0.188-2.029 | 0.427 |  | - | - | - |  | - | - | - |  | - | - | - |
| **Stage**  **(III/IV VS I/II)** | 2.665 | 1.905-3.730 | <0.001 |  | 2.84 | 2.024-3.982 | <0.001 |  | - | - | - |  | - | - | - |
| **Menopause status** |  |  |  |  |  |  |  |  |  |  |  |  |  |  |  |
| Pre (Ref) |  |  |  |  |  |  |  |  |  |  |  |  |  |  |  |
| Post | 1.279 | 0.842-1.943 | 0.248 |  | - | - | - |  | 1.685 | 1.431-1.983 | <0.001 |  | 0.633 | 0.501-0.799 | <0.001 |
| Peri | 0.906 | 0.457-1.794 | 0.776 |  | - | - | - |  | - | - | - |  | - | - | - |
| **Group**  **(Low VS High)** | 0.515 | 0.369-0.720 | <0.001 |  | 0.473 | 0.332-0.673 | <0.001 |  | 0.780 | 0.693-0.878 | <0.001 |  | 0.829 | 0.735-0.935 | <0.01 |
| **Chemotherapy**  **(Yes VS No)** | - | - | - |  | - | - | - |  | 1.228 | 1.057-1.427 | 0.007 |  | 1.741 | 1.450-2.091 | <0.001 |
| **Pathologic N**  **(>=N2 VS <=N1)** | - | - | - |  | - | - | - |  | 1.952 | 1.714-2.222 | <0.001 |  | 1.705 | 1.487-1.955 | <0.001 |
